# Application of Geopolymer in Stabilization/Solidification of Hazardous Pollutants: A Review

**DOI:** 10.3390/molecules27144570

**Published:** 2022-07-18

**Authors:** Quanzhi Tian, Yingchu Bai, Yinhai Pan, Changshuai Chen, Shuo Yao, Keiko Sasaki, Haijun Zhang

**Affiliations:** 1 National Engineering Research Center of Coal Preparation and Purification, China University of Mining and Technology, Xuzhou 221116, China; tianqz0502@cumt.edu.cn (Q.T.); ts20040066a31@cumt.edu.cn (Y.B.); ts20040089a31@cumt.edu.cn (Y.P.); tb19040018b2@cumt.edu.cn (C.C.); tb21040035b1@cumt.edu.cn (S.Y.); 2 School of Chemical Engineering and Technology, China University of Mining and Technology, Xuzhou 221116, China; 3 Department of Earth Resources Engineering, Kyushu University, Fukuoka 819-0395, Japan

**Keywords:** geopolymer, stabilization/solidification, cations, anions

## Abstract

Geopolymers, as a kind of inorganic polymer, possess excellent properties and have been broadly studied for the stabilization/solidification (S/S) of hazardous pollutants. Even though many reviews about geopolymers have been published, the summary of geopolymer-based S/S for various contaminants has not been well conducted. Therefore, the S/S of hazardous pollutants using geopolymers are comprehensively summarized in this review. Geopolymer-based S/S of typical cations, including Pb, Zn, Cd, Cs, Cu, Sr, Ni, etc., were involved and elucidated. The S/S mechanisms for cationic heavy metals were concluded, mainly including physical encapsulation, sorption, precipitation, and bonding with a silicate structure. In addition, compared to cationic ions, geopolymers have a poor immobilization ability on anions due to the repulsive effect between them, presenting a high leaching percentage. However, some anions, such as Se or As oxyanions, have been proved to exist in geopolymers through electrostatic interaction, which provides a direction to enhance the geopolymer-based S/S for anions. Besides, few reports about geopolymer-based S/S of organic pollutants have been published. Furthermore, the adsorbents of geopolymer-based composites designed and studied for the removal of hazardous pollutants from aqueous conditions are also briefly discussed. On the whole, this review will offer insights into geopolymer-based S/S technology. Furthermore, the challenges to geopolymer-based S/S technology outlined in this work are expected to be of direct relevance to the focus of future research.

## 1. Introduction

The sources of solid waste could be divided into several aspects, including industrial, residential, and commercial activities, etc. [1]. Since the last century, waste production has risen sharply as the world’s population has grown and gradually become more urban and affluent. It has been predicted that it will double again by 2025. Specifically, the global solid-waste generation will increase from more than 3.5 million tonnes per day in 2010 to more than 6 million tonnes per day in 2025 [2]. Solid-waste composition varies substantially with industries, technologies, locations, waste collections, etc. Generally, solid waste can be divided into several categories, including industrial waste (e.g., tailing, fly ash, slag, sludge, etc.), municipal waste (e.g., metal, paper, organic matter, etc.), and agricultural waste (e.g., rice husk, food waste, etc.) [3,4,5,6]. Normally, there are always certain amounts of hazardous pollutants, including heavy metals and organic contaminants, in solid waste [7,8]. Once this waste cannot be properly handled, serious air, water, and soil pollution occur. To solve the problems caused by solid waste, some rules, including resource recovery, waste reduction/minimization, safe disposal of waste, etc., have been proposed at the government level [9,10]. Resource recovery from solid waste is a complex procedure because the capital market is another important factor in the resource recovery process besides the advanced technologies. Currently, only 30% of the total waste material is involved in resource recovery globally [11]. In addition, waste minimization means reducing the volumes of hazardous waste for further landfilling or transportation. A typical example is the incineration of municipal solid waste, contributing to the reduction in waste mass and volume by 70% and 90%, respectively [12]. Therefore, the safe disposal of waste is still an extremely important way to treat solid waste.

The safe disposal of solid waste, especially hazardous waste, can be seen as the final and most vital step in effective waste management, and its main purpose is to prevent/reduce the motilities of pollutants in the waste [13]. Stabilization/solidification (S/S) is always adopted as the pretreatment process before landfilling or burying in the underground to achieve the safe disposal of hazardous waste [14]. Generally, the S/S process is to mix the solid waste with binding materials, including cement, asphalt, etc., to achieve good physical properties and, meanwhile, immobilize harmful components within solidified material [15,16]. Thus, binding material plays a critical role in the S/S performance for the specific waste. Ordinary Portland cement (OPC) is, of course, broadly adopted for the S/S process, due to its easy handling, low cost, and good S/S performance [17]. In addition, some other types of cement, such as magnesia-based cement, calcium aluminate cement, sulfate aluminate cement, etc., have been also adopted for the S/S of heavy metals [18,19,20,21,22]. However, cement-based S/S of waste is relatively fragile to physical and chemical degradation processes, which depends on factors, such as permeability, chemical and mineralogical composition, and microstructure [23]. Furthermore, the production of ordinary Portland cement consumes much energy and, meanwhile, leads to massive CO_2_ emissions [24,25,26]. Thus, more attention should be increasingly paid to developing environmentally friendly low-carbon green solidification materials.

Geopolymers are a kind of inorganic polymer consisting of SiO_4_ and AlO_4_ tetrahedrons connected by sharing the oxygen atoms [27]. They can be normally synthesized by low-temperature polycondensation of different materials, such as metakaolin and coal fly ash [28]. It has been viewed as a practical alternative to Portland cement because it exhibits better mechanical properties and durability, and has lower energy requirements and greenhouse gas emissions [29]. In recent years, geopolymers have gradually attracted much attention for S/S treatment. For the safe disposal of radioactive waste with high radioactivity, geopolymers, especially, can provide the potential for extremely long-term storage [30]. Over the past several decades, geopolymer technologies have been rapidly developed by academic researchers. There are also some reviews on the application of geopolymers from the perspectives of heavy metal sorption [31,32] and adsorbents for wastewater [33]. However, the sorption process can be seen as a part of the S/S process, and there is still a significant difference between them. By now, there are few reviews reported about the geopolymer-based S/S process. Thus, this review firstly attempts to comprehensively present the S/S of hazardous pollutants, including heavy metals and organic contaminants, using geopolymer as the binding material. This paper will contribute to the further understanding of the geopolymer-based S/S process of hazardous pollutants.

## 2. Geopolymer

Since Prof. Joseph Davidovits introduced the terminology geopolymer in the 1970s [34], it has been developed for more than thirty years. Geopolymer is most commonly referred to inorganic aluminosilicate based on geological materials, which can react with an alkaline solution to form a binder through a polycondensation reaction [35]. There is also another way to use an acidic phosphate component or phosphoric acid as the activator for the complex acid-based reaction to initiate. The polymerization occurs between the Al–O layer in the aluminosilicate and P–O tetrahedron in the activator, and the obtained solid can also be called chemically bonded phosphate ceramics [4,36]. In addition, the borate can be also substituted for the silicate in aluminosilicate to participate in the geopolymerization reaction [37]. Generally, geopolymer can be formed through the geopolymarization process, mainly including dissolution, nucleation, oligomerization, and polymerization [38]. Taking an alkali-activated geopolymer as an example, the aluminate and silicate can be dissolved from raw material under alkali conditions. Then, the dissolved Al and Si tetrahedrons go through gelation, reorganization, and polymerization to form the hardened geopolymer paste [39]. Because of the negative charges generated from Al tetrahedrons, cations, such as Na^+^ and K^+^, are needed to achieve the charge balance. In addition, geopolymer is a kind of amorphous substance with a zeolite-like structure, and alkali metals (Na^+^ or K^+^) can be exchanged with other cations, such as Cs^+^, Cd^2+^, Pb^2+^, etc. [31].

Based on the aluminosilicate sources, geopolymers can be divided into several types, such as fly ash-based geopolymer, metakaolin-based geopolymer, slag-based geopolymer, and mine tailing-based geopolymer, etc. [40,41]. Different aluminosilicate sources possess various chemical activities. Therefore, the properties of generated geopolymers, such as mechanical performance, thermal behavior, durability, etc., have a close relationship with aluminosilicate sources. It is widely accepted that amorphous phases in the aluminosilicate source are the main reactive components during the geopolymerization process, and most of the crystalline phases are hardly dissolved during the reaction [42]. Thus, thermal activation was proposed as an effective method to modify the physicochemical properties of materials. During thermal treatment, phase transformations occur in the material, resulting from the loss of volatile components and reorganization of atomic structures [43]. For example, the dehydroxylation of kaolinite at temperatures between 700 and 900 °C can convert its crystalline structure to an amorphous substance, which can be activated by alkaline solutions [44]. However, there is no change for some minerals, such as quartz, and mullite after thermal treatment. Alkaline fusion was proposed to increase the geopolymeric reactivity of materials. The structures of quartz and mullite are decomposed by the calcination of the mixture of raw material and alkaline, then promoting the dissolution of Si and Al species [43]. In addition, mechanical activations, including grinding, comminution, etc., have also been put forward to increase the specific surface areas and reaction sites [45]. On the other hand, NaOH or KOH and Na_2_SiO_3_ or K_2_SiO_3_ solutions are commonly used as the activators for geopolymer synthesis [46,47]. Soluble silicate has a significant effect on the properties of geopolymer. Generally, the geopolymer pastes activated by Na_2_SiO_3_ solution always have better mechanical strength than that obtained from NaOH activation [46,47].

There are some unique features for geopolymer. From the view of the structure, geopolymer has an amorphous structure, which is always regarded as the precursor of zeolite due to their similar composition [48]. The differences between the synthesis of geopolymer and zeolite mainly refer to reaction conditions, including Si/Al molar ratio of raw material, liquid/solid ratio, and ambient temperature. For instance, zeolite can normally be formed under the condition of an H_2_O/SiO_2_ molar ratio of 10–100 and OH^–^/SiO_2_ of 2–20 [49]. However, the reaction condition of H_2_O/SiO_2_ molar ratio of 2–10 and OH^–^/SiO_2_ of 0.1–0.5 is employed for geopolymer [50]. At a certain condition, the crystal minerals, such as pollucite, nepheline, etc., can be generated in the final product after thermal treatment is conducted to the geopolymer [51,52]. Another important point that should be noted from a view of macroscopic property is that a dense and compact structure can be formed for geopolymer [53]. This should be the principal consideration as to why geopolymer can be called man-made stone. It has been proved that some additives, such as calcium oxide, magnesium oxide, etc., could influence the compactness of geopolymer paste [54,55]. This provides an effective method for the control of geopolymer compactness, since higher compactness of geopolymer, to a large extent, can reduce the leaching amount of pollutants from S/S paste.

## 3. Geopolymer-Based S/S Process

### 3.1. S/S of Heavy Metals

Heavy metal pollution has become a severe problem in many parts of the world, mainly caused by anthropogenic activities, including mining, smelting, coal combustion, etc. [56]. Specifically, it is precisely because of the improper disposal of the waste produced from these anthropogenic activities. Heavy metals can be converted, persistent and irreversible in the environment because heavy metals cannot be decomposed, but they can exist in various chemical associations. Normally, heavy metals can be divided into cationic and anionic metals and both of them can cause serious pollution to the environment. A summary on the S/S of typical heavy metals in geopolymers is presented in Table 1. Due to their different immobilization mechanisms, a statement of the S/S process for cationic and anionic metals was described in detail.

#### 3.1.1. S/S of Cationic Metals

##### Pb

**Figure 1:**
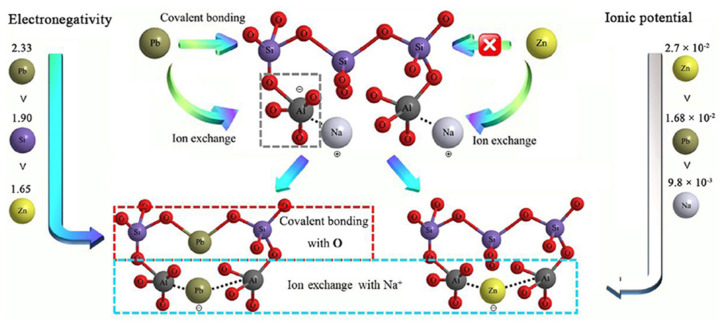
Associations of Pb and Zn in geopolymer [76].

The applications of geopolymers in the S/S of Pb have been widely reported. Chen et al. [57] adopted coal gasification fly ash to synthesize geopolymer for the immobilization of Pb. Most Pb was remained in the aluminosilicate structure of the geopolymer. Hu et al. [58] prepared geopolymer using rare earth tailing and metakaolin, showing extraordinary S/S performance of Pb^2+^. The Pb^2+^ was participated in the polycondensation of the Si/Al gel phase and formed PbO inside the network of the geopolymer. Furthermore, Guo et al. [59] utilized coal fly ash to synthesize geopolymer for the immobilization of Pb and compared the immobilization efficiency of geopolymer to Pb compounds, such as PbO, PbSO_4,_ and PbS. The results indicated that the immobilization limit of Pb content is approximately 4% for PbO and PbSO_4_, and 8% for PbS. Except for common waste, such as fly ash, tailings, and drinking water, treatment residuals were also adopted as the raw materials of geopolymer synthesis for Pb immobilization [73,74]. There are also reports on geopolymer synthesis using lead–zinc smelting slags and Pb in slags can be well solidified through physical encapsulation and chemical associations [75,76]. As a whole, it is commonly recognized that Pb^2+^ could attack the SiO_4_ tetrahedron structure and form covalent bonds in the Pb–O structure (Figure 1), while, due to the negative charge of Al tetrahedrons and higher ion potential of Pb^2+^, Pb^2+^ can exchange with Na^+^ or K^+^ in the structure of the geopolymer [77]. However, Pb^2+^ would precipitate under an alkaline medium. Therefore, ion exchange of Pb^2+^ with Na^+^ or K^+^ could be the main interaction of sorption using geopolymer as an adsorbent. It should be noted that sorption can be included in the stabilization process. There are so many publications about the adsorption of Pb^2+^ using geopolymers synthesized from metakaolin [78], fly ash [79,80,81], slag [82], foundry dust [83], mine tailings [84], biofuel ash [85], and red mud [86]. To further improve the adsorption efficiencies, zeolite-based adsorbents transformed from geopolymers were developed and achieved better adsorption performance of Pb^2+^ [87,88,89,90,91]. Furthermore, geopolymer-based composites, such as geopolymer–alginate–chitosan composites [92] and porous microsphere/geopolymers [93], were designed and used for the adsorption of Pb^2+^. In addition to alkali-activated geopolymers, an acidic phosphoric-based geopolymer was adopted for the S/S of Pb. It was shown that the acidic phosphoric-based geopolymer has a better S/S performance of Pb^2+^ than that of alkali-activated geopolymers under acidic conditions [94]. Pb^2+^ can react with ${PO}_{4}^{2-}$ to form stable compounds, including Pb_3_(PO_4_)_2_ and PbHPO_4_, which are proposed as the main reaction mechanisms.

##### Zn

Zinc is also a very communal pollutant in the environment and the improper disposal of waste, such as lead–zinc slags, mine tailings, etc., can cause serious pollution [95]. Nath [96] utilized zinc slag and fly ash to prepare geopolymers and the leaching of Zn from geopolymers containing 40–80% slags is within the permissible limit. Sun et al. [60] used sludge residue to synthesize geopolymer and achieved a high-efficient immobilization of the unstable forms of Zn. It is also reported that the immobilization rate of more than 99% for Zn can be achieved by geopolymer-based S/S [61]. Similarly, the alkaline condition of the geopolymer plays an important role in the S/S of Zn. However, to some extent, Zn can be dissolved in alkaline conditions [97]. Wan et al. [62] reported that Zn can be stabilized in geopolymers through physical encapsulation and adsorption of leached Zn^2+^ by the geopolymer. Wang et al. [98] also proposed that Zn^2+^ can partially replace Na^+^/K^+^ balanced with the negative charge of an Al tetrahedron in geopolymer structure (Figure 1). Therefore, the reaction mechanisms of geopolymer-based S/S of Zn can be summarized as physical encapsulation and electrostatic adsorption [99]. On the other hand, compared to the solidification of Zn using geopolymer, there are more reports about the adsorption of Zn^2+^ using geopolymer-based adsorbents, such as natural volcanic tuff-based geopolymers [100], metakaolin-based geopolymers [101], fly-ash-based geopolymers [102], hollow gangue microsphere/geopolymer composite [93], clay-fly-ash-based geopolymer [103], etc. As a whole, Zn and its compounds possess the properties of amphoteric metal, and the specific associations of Zn in geopolymers should be further explored.

##### Cd

**Figure 2:**
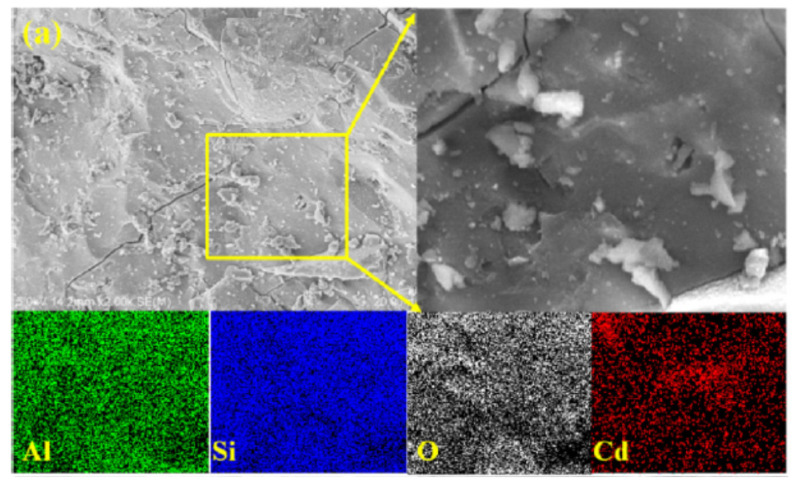
Distribution of Cd in geopolymer [74].

Cadmium, as one of the most toxic heavy metals, can cause serious disorders for humans, such as heart disease, cancer, or diabetes [104]. Zhang et al. [105] utilized fly ash-based geopolymer to immobilize Cd and found that Cd immobilization is mainly related to the solubility of a hydroxide phase and can be effective at high pH. Furthermore, Muhammad et al. [63] observed that a higher leaching amount of Cd occurred in an acidic medium from a ground-granulated blast furnace slag and fly-ash-based geopolymers. Zheng et al. [106] suggested that the crystalline phase containing Cd was not produced in geopolymer. Ji et al. [64,74] indicated that the main form of Cd should be a divalent state linked as Al–O–Cd in the drinking water treatment residue-based geopolymer. Wang et al. [107] further pointed out that Cd^2+^ was considered to balance the negative charge of Al tetrahedrons in the geopolymer framework. However, Wang et al. [65] proposed that the formation of Cd(OH)_2_ was the main solidification mechanism of Cd^2+^ in geopolymers. By now, it has not been certified that these two kinds of associations are included in the geopolymer S/S of Cd, which might be largely influenced by the concentration of Cd. As a whole, the distribution of Cd can be well overlapped with Al and Si (Figure 2). El-eswed et al. [108] confirmed that Cd cannot replace Si in the geopolymer framework. Anyway, geopolymers possess excellent immobilization performance of Cd^2+^ through physical encapsulation and chemical stabilization [109]. Being similar to the case of Pb^2+^, geopolymer-based adsorbents, such as magnetic geopolymer [110], dolochar ash-based geopolymer [111], and zeolite-based geopolymer [112], etc., have been designed and studied for the adsorption of Cd^2+^. A large adsorption amount of Cd^2+^ can be achieved by geopolymer-based adsorbents. Ion exchange should also be the main reaction mechanism for the Cd^2+^ sorption process onto geopolymer [77].

##### Cs

Cs is one of the alkali metals and possesses good mobilities in both acidic and alkali conditions. ^137^Cs, as one of the most hazardous radionuclides, possesses a long half-life (30 years) and strong radioactivity [113]. Compared to ordinary Portland cement (OPC), geopolymer shows better S/S performance of Cs due to their excellent properties. Kozai et al. [114] compared geopolymer-based S/S with cement (OPC)-based S/S and indicated that less than 1% Cs^+^ was released from the geopolymer, while more than 30% was leached from OPC. Tian et al. [115] also found that less Cs^+^ was leached out from geopolymer where Cs-zeolite was solidified. Li et al. [66] solidified Cs^+^ in a fly-ash-based geopolymer and less Cs^+^ was leached out from geopolymer blocks under acidic or saline conditions. Furthermore, He et al. [116] found that Na-based geopolymers presented a lower leaching rate of Cs^+^ than K-based geopolymers, and higher temperature and saline solution can accelerate the leaching of Cs^+^. Since Cs^+^ can replace Na^+^ or K^+^ in the geopolymer structure, ion exchange is the main mechanism for the S/S of Cs^+^ in geopolymers, which was firstly proposed by Bortnovsky et al. [117]. To further improve the S/S efficiency of Cs^+^ in geopolymer, a series of strategies have been proposed. Ofer-Rozovsky et al. [118] and Haddad et al. [119] prepared a series of low-Si geopolymers for the S/S of Cs, and crystal minerals, such as zeolite A, zeolite F, zeolite F, feldspathoid, etc., can be formed from the amorphous structure of the geopolymer, contributing to the enhanced immobilization of Cs^+^.

**Figure 3:**
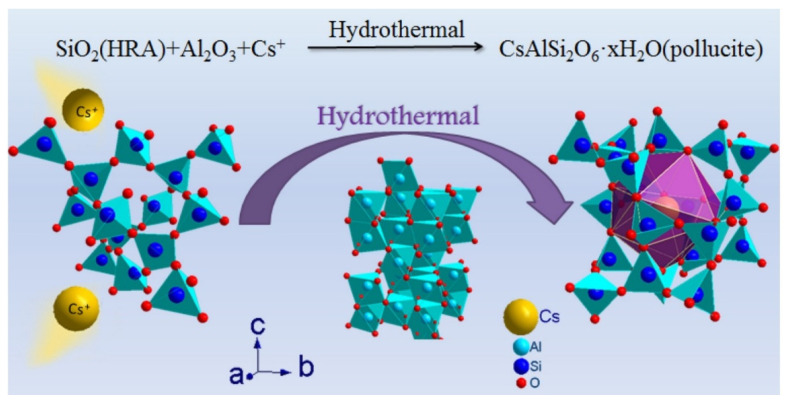
S/S of Cs in geopolymer under thermal treatment [132].

Thermal treatment has been proposed as an effective method to further improve the S/S efficiency of Cs (Figure 3). Under high temperatures, geopolymer can be viewed as precursor for the formation of ceramic, and meanwhile, pollucite has been regarded as one of the most stable phases for the safe disposal of Cs [120]. It has been reported that geopolymer containing Cs^+^ could be gradually crystallized into pollucite upon heating above 900 °C [121]. Chlique et al. [122] pointed out that a higher concentration of Cs^+^ in geopolymer tends to reduce the amorphous phase and improve the quantification of pollucite and nepheline. He et al. [123] found pollucite can be formed from Cs-based geopolymers synthesized from synthetic metakaolin at a lower temperature (800~1000 °C). Furthermore, He et al. [67] obtained pollucite with excellent S/S performance of Cs from geopolymer calcined at a low temperature (<1000 °C) via alkali metal ion doping and optimizing the Na/Cs ratio. Chen et al. [124] proposed a hybrid hydrothermal-sintering process including a 200 °C hydrothermal process and an additional 1200 °C sintering process, which can effectively lower the amount of Cs lost to volatilization. To improve the efficiencies of pollucite formation, Xiang et al. [125] adopted rapid microwave sintering to prepare Cs-defined ceramics, which can be formed at below 1100 °C within 30 min. He et al. [113] found that pollucite can be formed at approximately 700 °C with the addition of 7.5 wt% B_2_O_3_. Liu et al. [126] investigated the effect of sodium salts on the hydrothermal process (<200 °C) of a volcanic-ash-based geopolymer and concluded that the introduction of CsOH·H_2_O can promote the formation of pollucite. Concerning the structure of the Cs-geopolymer and its thermal product, the pair distribution function (PDF) has always been adopted to analyze their microstructures [127]. On the other hand, it should be stated that geopolymer-based adsorbents, such as layered double hydroxide/geopolymers [128], phosphoric-acid-based geopolymers [129], graphene oxide/geopolymers [130], geopolymer foams [131], etc., have been well developed for the adsorption of Cs^+^ in aqueous conditions.

##### Other Cations

In addition to the cationic metals discussed above, there are still other metals, such as Sr, Cu, Ni, V, Co, U, etc., which have also been targets solidified in geopolymers. ^90^Sr is also one of the radionuclides that can be generated in the fusion process, as well as ^137^Cs. Liu et al. [68] investigated the immobilization of simulated ^90^Sr in a fly-ash-slag-metakaolin-based geopolymer, and the cumulative fraction leaching rate of ^90^Sr is much lower than that of cement. Jang et al. [69] indicated that the diffusivity of soluble Sr^2+^ ions was highly correlated with the critical pore diameter of the binder. Therefore, geopolymers can better retard the diffusion of nuclides due to their compact structure. From the view of the structure, Walkley et al. [133] found that both Sr^2+^ and Ca^2+^ can induce the same structural change in the gels and partially Sr-substituted zeolite A was formed with the gels cured at 80 °C. Furthermore, Li et al. [134] proposed that the distribution of Sr^2+^ had a closed relationship with Ca^2+^ in geopolymer. Thus, an effective S/S method for Sr can be developed with the consideration of Ca properties. The formation of a nepheline structure in geopolymer at 1200 °C for the blocking of Sr was proposed, which largely reduced the leaching rate in the deionized water [135]. In most cases, the radioactive Sr^2+^ was firstly adsorbed onto adsorbents, such as zeolite A [136], clinoptilolite [137], and titanate ion exchangers [138,139], etc., which were then encapsulated in geopolymers, showing great S/S performance.

In addition, Zhang et al. [140,141] verified that fly ash- or slag-based geopolymers could effectively immobilize Cu and achieve an immobilization efficiency of higher than 90% under the concentration of Cu in a range of 0.1–0.3%. El-eswed [108] proposed that Cu can replace Si in the geopolymer structure, being similar to Pb. Moreover, the leaching of Cu was always evaluated in terms of geopolymers synthesized from fly ash and industrial sludge [142], mine tailing [143], lead–zinc smelting slag [75], sludge incineration residue [144], red mud, and municipal solid waste incineration fly ash (MSWI) [145,146,147], melting slag of MSWI and bottom ash [148], electrolytic manganese residue [149], electric arc furnace dust [150], etc. Importantly, the leaching concentration of Cu can be satisfied with related standards. This can also be found in the cases of Ni [151,152,153,154] and V [155,156,157,158] immobilizations using geopolymers. Compared to the above-mentioned metals, there are only a small number of reports about Co, U, etc. Yu et al. [70] utilized Mn-slag to synthesize geopolymer for the S/S of Co and found that divalent Co was oxidized to trivalent Co in the matrix, contributing to higher solidification capacity. Zhou et al. [159] immobilized U-contaminated soils using a coal gangue-based geopolymer and achieved a fixation efficiency of U up to approximately 77%. Furthermore, Li et al. [160] added nano-hydroxyapatite into geopolymer to enhance the S/S performance of U and the leaching concentration of U from the solidified body with an additive was low to 15 ppm. Chen et al. [161] designed a thermal method for stabilizing U(VI) in red mud, contributing to a successful solidification. As a whole, geopolymers can possess good S/S ability for cationic pollutants.

**Figure 4:**
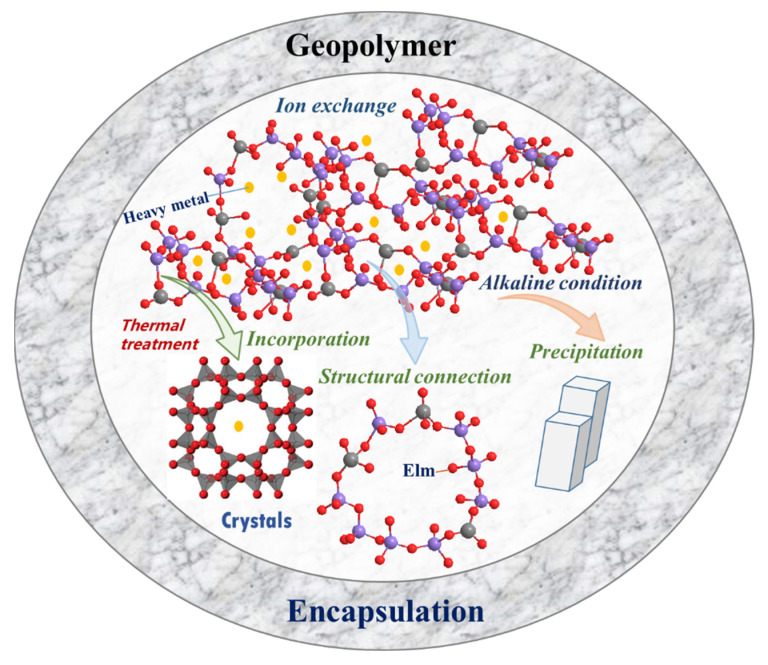
S/S mechanisms of heavy metals in geopolymer.

The main geopolymer-based S/S mechanisms for cations are concluded, as shown in Figure 4. Physical encapsulation is the basic function of geopolymer, which can achieve high mechanical strength and compact structure, protecting immobilized waste from contact with solutions. In addition, the negative charges produced by Al tetrahedrons can permit cation exchange with Na^+^ or K^+^. Under thermal treatment, the amorphous structure of geopolymer can be transformed into crystals, chemically enhancing the S/S performance. In addition, during the geopolymerization process, some cations can be replaced with Al and be linked with Si directly. On the other hand, precipitation of cations is also another important role for the geopolymer-based S/S.

#### 3.1.2. S/S of Anionic Metals

##### Cr

Chromium can exist in the form of both cationic ions (Cr^3+^) and anionic ions (Cr$O_{4}^{2-}$ and Cr_2_$O_{7}^{2-}$). Importantly, Cr(VI) possesses better mobilities and higher toxicity than Cr(III) [162]. Therefore, the S/S of Cr was discussed in the current section. Al-Mashqbeh et al. [163] utilized metakaolin-based geopolymer to immobilize the inorganic anions (Cr_2_$O_{7}^{2-}$, ${MnO}_{4}^{-}$, and Fe${\left(CN\right)}_{6}^{3-}$) and concluded that geopolymer had limited capacity for the immobilization of metal anions. Nikolić et al. [71] employed fly ash-based geopolymer to immobilize Cr(VI) and found that geopolymer pastes containing Cr (0.5–2.0%) cannot meet the requirement of landfills due to the high leaching concentration. Moreover, Muhammad et al. [63] used polycarboxylate superplasticizer to enhance the compressive strength. However, a higher leaching amount of Cr(VI) can be observed in an acidic medium. Thus, the reduction of Cr(VI) to Cr(III) using zero-valent iron [164,165], ferrous salts [166,167], and sulfide [168,169] has been proposed as an important strategy for the higher geopolymer-based S/S ability. It has been proved that the detoxification of Cr(VI) before solidification is more efficient, as compared to the simultaneous reduction in and solidification of Cr(VI) in the geopolymer. Then, the Cr(III) reduced from Cr(VI) can be stabilized through sorption and ion exchange in the geopolymer, which is similar to other cations [170]. Based on the mechanism, some adsorbents, such as green-rust-functionalized geopolymers [171], geopolymer–zeolite composite membranes [172], and organically modified geopolymers [173], etc., were designed and applied in the removal of Cr(VI) from solutions. On the other side, Wei et al. [174] synthesized a Cr(VI)-added geopolymer treated by hydrothermal processing, and the leaching of Cr was far below the available limit, suggesting that hydrothermal processing can be a potential candidate technique for the disposal of Cr.

##### Se

**Figure 5:**
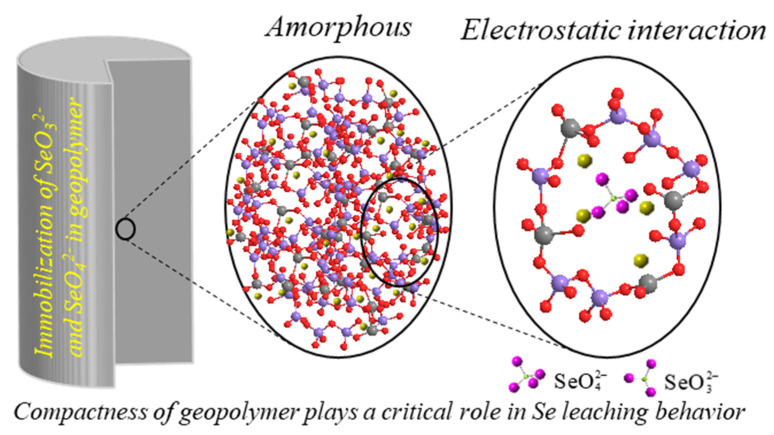
Association of Se oxyanions in geopolymer [72].

Strictly speaking, selenium is not a metal element. ^79^Se is one of the fission products in nuclear reactors and it possesses an extremely long half-life of approximately 1.11 × 10^6^ [175,176,177]. Serious injuries to plants, animals, and even humans would be caused once ^79^Se is unintentionally released into the environment (e.g., the Fukushima accident), because of its strong radioactivity. Generally, Se can exist in several oxidation states, including selenide (–II), elemental Se (0), selenite (IV), selenate (VI), etc. Among them, ${\mathrm{SeO}}_{3}^{2-}$ and ${\mathrm{SeO}}_{4}^{2-}$ are the dominant species in water environments, with high mobility and transportability [178]. However, there are only a few studies about the S/S of Se in geopolymers. It has been reported that more Se was leached out from geopolymers synthesized by coal fly ash and slag [158], coal fly ash and metakaolin [157], spent aluminate, and fly ash [179], etc. Tian et al. [72] reported that sodium silicate-activated geopolymers can substantially reduce the Se leaching amount compared to sodium hydroxide-activated geopolymers, and also proposed that electrostatic interaction is the main association of ${\mathrm{SeO}}_{3}^{2-}$ and ${\mathrm{SeO}}_{4}^{2-}$ in geopolymers (Figure 5). The cations, such as Na^+^, bridged the negative charge produced by the Al tetrahedron and oxyanion, which was certified by the sequential study [180]. On the other hand, compactness plays a vital role in the Se leaching from geopolymers.

##### Other Anions

There are always geopolymer-based adsorbents developed for the removal of anions, such as I^−^ [181], F^–^ [182,183], Se oxyanions [128,184], As oxyanion [185], P oxyanion [186], S oxyanion [187], Sb oxyanion [188], etc. In most cases, geopolymers are adopted as the matrix, and sorption active sites are loaded through ion exchange, precipitation, etc., Compared to cationic metals, there are few studies reported about the S/S of anionic species using geopolymers, except for the above-mentioned elements in detail. High leaching amounts of anions always occurred in most cases due to the negative charge repulsion. Al-Mashqben et al. [163] also indicated that geopolymer has limited immobilization capacity for the encapsulation of heavy metal anions, including ${\mathrm{MnO}}_{4}^{-}$ and Fe${\left(\mathrm{CN}\right)}_{6}^{3-}$. Arsenic is a typical element belonging to metalloids, possessing hypertoxicity. It has been stated that arenite can also be associated in the geopolymer structure by electrostatic interaction [180]. This is similar to the case of Se oxyanions. However, arsenate ions tend to recrystallize into the arsenate compound (Na_3.25_(OH)_0.25_(H_2_O)_12_(AsO_4_)) in geopolymers [180].

### 3.2. S/S of Organic Pollutants

Generally, organic waste is always burned to reduce the volume. It has been proved that organic compounds are poorly retained in inorganic materials, such as ordinary Portland cement [189]. Therefore, there are few studies about the S/S of organic pollutants in geopolymers now. Only several reports were published on the preparation of geopolymer-based photocatalysts, including graphene/fly-ash-based geopolymers [190], TiO_2_-doped zeolite/geopolymers [191], Cu (I) oxide, and titanium dioxide/geopolymers [192], for the degradation of organic pollutants or the adsorbents/membrane, including magnetic geopolymers [193], defective analcime/geopolymer membranes [194], geopolymer/alginate [195], for the removal of organic pollutants from the aqueous conditions.

## 4. Future Perspectives

Even though geopolymers, as a kind of promising material, have been widely studied in the S/S of hazardous pollutants, there are still some challenges that require further exploration. First of all, geopolymer possesses a negative charge provided by Al tetrahedrons, showing a repulsive effect on anionic ions. Although it has been certified that anionic species, such as the oxyanions of Se and As, can exist in the three-dimensional structure of geopolymer through electrostatic interaction, leaching amounts of them are still higher than the relevant standards. More effective methods should be developed to enhance the ability of geopolymers for the S/S of anionic species. In addition, geopolymers always show good S/S ability for cationic ions. Thermal treatment (≥1000 °C) is always used to improve its S/S efficiency, which consumes plenty of energy. Under the background of carbon neutrality, it is imperative to find alternative ways to improve the S/S efficiency with a lower-temperature thermal treatment. In addition, the S/S of hazardous pollutants, especially radionuclides, normally require long-term stability. Nevertheless, less attention was paid to the properties of the solidified body in the long-term run. On the other hand, most publications are largely concerned about the leaching behavior of immobilized pollutants from blocks. There are few works conducted to elucidate the influence of encapsulated pollutants on the mechanical properties of geopolymers. Therefore, even though so many studies have been reported, geopolymer-based S/S technology requires more effort to make great achievements.

## 5. Conclusions

Geopolymers, as a kind of novel inorganic polymers, possess excellent properties of high mechanical strength, good durability, chemical resistance, etc. Furthermore, solid waste, such as coal fly ash and slags, can be adopted as raw materials for geopolymer synthesis, pointing out that these wastes can be safely disposed of through chemical transformation, or can be used for S/S of other hazardous waste. Therefore, geopolymer shows its unique advantages for the immobilization of hazardous pollutants. Currently, geopolymers have been widely studied for the S/S of hazardous pollutants, which are divided into heavy metals (cations and anions) and organic pollutants. The S/S of cationic metals, including Pb, Zn, Cd, Cs, Sr, Cu, Co, Ni, U, and V, have been examined by geopolymers synthesized from metakaolin, fly ash, slags, solid residues, etc. The immobilization process for these metals is mainly dependent on the mechanisms of physical encapsulation, sorption, precipitation, and bonding with silicate structures. In addition, geopolymers have also been adopted for the S/S of anionic pollutants, such as Se oxyanions, As oxyanions, Cr oxyanions, etc. It has been verified that Se or As oxyanions can be associated in geopolymer through electrostatic interaction, even though the leaching amounts are still higher compared to cationic metals. There are few reports about the geopolymer-based S/S of organic pollutants. Besides, the development of adsorbents for heavy metals and some organic pollutants is always a hot topic in research. Furthermore, the challenges to geopolymer-based S/S technology outlined in this work are expected to be of direct relevance to the focus of future research. As a whole, this review will offer insights into the use of geopolymers as S/S materials for various pollutants.

## Figures and Tables

**Table 1:** Recent studies about S/S of heavy metals using geopolymers.

Materials	Heavy Metal Species (Content)	Curing Condition	Leaching Conditions or Methods	Leaching Concentration (mg·L^−1^)	Immobilization Efficiency	Reference
Coal gasification fly ash Metakaolin Steel slag	Pb (4 wt%) Zn (4 wt%) Cr (0.5 wt%)	Cured at room temperature for 3 and 7 days.	EPA Method 1311	–	Pb (93.12–99.29%) Zn (93.85–96.74%) Cr (95.44–99.45%)	[57]
Rare earth tailing Metakaolin	Pb (0.2 wt% to 1 wt%)	Cured at 60 °C for 8 h and then at room temperature for another 1, 3, and 7 d.	EPA Method 1311	Pb (<0.1) Ba (<0.4)	Pb (>95%) Ba (>95%)	[58]
Coal fly ash	Pb (1 wt% to 8 wt%)	Cured at 85 °C for 24 h and then at room temperature for another 7 days.	EPA Method 1311	Pb (6–116)	Pb (>98.9%)	[59]
Sludge residue	Zn (2726 mg/kg) Cu (1077 mg/kg)	Cured at room temperature for 7 days.	EPA Method 1311	Zn (1.33) Cu (0.02)	Zn (>95%) Cu (>95%)	[60]
Municipal solid waste incineration	Pb (2249 mg/kg) Zn (6368 mg/kg) Cd (282 mg/kg)	Cured at the temperature of 20 ± 2 °C and humidity higher than 90% for 7, 14, and 28 d.	HJT300-2007	Pb (0.085) Zn (0.766) Cd (0.054)	Pb (>99%) Zn (>99%) Cd (>99%)	[61]
Zinc mine tailing Metakaolin	Zn (2.1%)	Cured at 60 °C for 6 h and then cured at room temperature for 7 days.	EPA Method 1311	Zn (2.77)	Zn (>99.09%)	[62]
Fly Ash Ground Granulated Blast Furnace Slag	Pb (0.1–0.5%) Cd (0.1–0.5%) Cr (0.1–0.5%)	Cured at 70 °C for 24 h and then Curing at room temperature for 28 days.	HJ/T 300–2007	–	Pb (91–99.99%) Cd (99.13–99.69%) Cr (91–97%)	[63]
Drinking water treatment residue municipal waste incineration bottom ash	Pb (1–4%) Cd (1–4%) Zn (1–4%)	Cured at 80 °C for 8 h and then at room temperature for another 7, 14, and 28 days.	EPA Method 1311	Pb (<10) Cd (<12) Zn (<3)	Pb (>99.43%) Cd (>99.43%) Zn (>99.43%)	[64]
Fly ash Ground granulated blast-furnace slag	Pb (2%) Cd (2%)	Cured at a temperature of 20 ± 3 °C and relative humidity of 95% for 28 days.	EPA Method 1311	Pb (0.14 to 2.55) Cd (<1)	Pb (92.98–94.67%) Cd (>99.943)	[65]
Fly ash	Cs (2%)	Cured at 60 ± 0.5 °C for 28 days.	pH = 1 H_2_SO_4_ solution or 5% (wt) MgSO_4_ solution	–	Cs (<0.5%)	[66]
Metakaolin	Cs (33.37 wt%)	Cured at 60 °C for 48 h, and calcined at low temperature (≤1000 °C) for 2 h.	EPA Method 1311 ANSI/ANS 16.1-2003	–	Leaching rate: 2.51 × 10^−4^ g m^−2^ d^−1^	[67]
Fly ash Slag Metakaolin	Sr (1, 3, 5, 7, and 9 wt%)	Cured at 25 ± 1°C for 24 h and calcined at the temperature (≤1000 °C) for 2 h.	Deionized water	–	Leaching rate: <2 × 10^−3^ g m^−2^ d^−1^	[68]
Fly ash	Cs (10 g/L) Sr (10 g/L)	Cured at 60 °C for 24 h and then at room temperature for four weeks.	ANSI/ANS-16.1-2003	–	Cs (95.95–96.79%) Sr (>99.96%)	[69]
Mn slag Metakaolin	Co (1.13 wt%)	Cured at a temperature of 25 ± 0.5 °C and relative humidity of 90% for 30 days.	EPA Method 1311	–	Co (>99.65%)	[70]
Fly ash	Cr (0.5–2%)	Cured at room temperature and relative humidity of 90 ± 5% for 28 days.	SRPS EN 12457-2	Cr (3.78)	–	[71]
Metakaolin	Se (2%)	Cured at room temperature and relative humidity of 93 ± 2% for 28 days.	EPA Method 1311	–	Se (24.15–93.74%)	[72]

Note: Leaching procedures can be referred to as the standard documents of each method.

## Data Availability

The data presented in this study are openly available in ScienceDirect at https://doi.org/10.1016/j.jhazmat.2019.121994, https://doi.org/10.1016/j.jhazmat.2019.121290, https://doi.org/10.1016/j.envpol.2021.118509, https://doi.org/10.1016/j.jhazmat.2015.12.024, and reference number [72,74,76,132]. In addition, the data (Figure 4) presented in this study are available on request from the corresponding author.
